# The developmental trajectory of diurnal cortisol in autistic and neurotypical youth

**DOI:** 10.1017/S0954579423000810

**Published:** 2023-07-12

**Authors:** Blythe A. Corbett, Trey McGonigle, Rachael A. Muscatello, Jinyuan Liu, Simon Vandekar

**Affiliations:** 1 Department of Psychiatry and Behavioral Sciences, Vanderbilt University Medical Center, Nashville, TN, USA; 2 Vanderbilt Kennedy Center, Vanderbilt University Medical Center, Nashville, TN, USA; 3 Department of Psychology, Vanderbilt University, Nashville, TN, USA; 4 Department of Biostatistics, Vanderbilt University Medical Center, Nashville, TN, USA

**Keywords:** Autism, Puberty, Diurnal, Cortisol

## Abstract

Increasing age and puberty affect the hypothalamic pituitary adrenal (HPA) axis maturation, which is likely associated with an increase in environmental demands (e.g., social) and vulnerability for the onset of psychiatric conditions (e.g., depression). There is limited research as to whether such patterns are consonant in youth with autism spectrum disorder (ASD), a condition marked by social challenges, dysregulation of the HPA axis, and higher rates of depression setting the stage for enhanced vulnerability during this developmental period.

The current study interrogated diurnal cortisol by examining (1) cortisol expression longitudinally over the pubertal transition between autistic and neurotypical youth, (2) the trajectory of diurnal cortisol and the unique contributions of age vs. puberty, and (3) potential sex differences. As hypothesized, results indicate autistic compared to typically developing youth demonstrate a shallower diurnal slope and elevated evening cortisol. These differences were in the context of higher cortisol and flatter rhythms based on age and pubertal development. Also, sex-based differences emerged such that females in both groups had higher cortisol, flatter slopes, and higher evening cortisol than males. The results show that despite the trait-like stability of diurnal cortisol, HPA maturation is impacted by age, puberty, sex, as well as an ASD diagnosis.

## Introduction

The dynamic adolescent period is marked by significant physiological changes in the regulation and responsivity of the hypothalamic–pituitary–adrenal (HPA) axis resulting in elevations in diurnal basal cortisol levels (Barra et al., 2015) and higher cortisol in response to perceived stressors (Gunnar et al., 2009). Cortisol is a glucocorticoid secreted by the adrenal cortices and the final hormonal output of the HPA axis in humans. Cortisol exhibits a characteristic diurnal rhythm with peak concentrations in the morning and nadir concentrations in the evening. Specifically, cortisol rises immediately after waking followed by an increase 30–60 min post-waking, then a sharp decline with a relative plateau in the afternoon and the lowest levels in the evening. The cortisol awakening response (CAR) refers to the sharp increase of 38–75% of blood levels of cortisol (Pruessner et al., 1997; Wust et al., 2000). The CAR is present in the majority of adults but less consistent in adolescents and shows significant intra- and inter-variability (Almeida et al., 2009; Platje et al., 2013; Ross et al., 2014). The rise of cortisol in the morning is thought to be related to the act of waking (Fries et al., 2009; Wilhelm et al., 2007); thereby, preparing the person for enhanced physiological arousal to meet the demands of the day. While cortisol levels fluctuate in a circadian rhythm (Knutsson et al., 1997), there is a trait-like stability explaining an estimated 72% of the variance that is distinct from response to state-based events or stressors, and this trait-like component is largely stable over time (Shirtcliff et al., 2012).

Adolescence refers to the developmental transition shaped by psychological and social experiences measured by age (Spear, 2000; Steinberg, 2005) and generally defined as *early* (10–14 years), *middle* (15–17 years), and *late* adolescence/young adulthood (17–24). Puberty explicitly refers to biological maturation initiated via activation of the hypothalamic–pituitary–gonadal (HPG) axis resulting in significant changes in cognitive, emotional, and physiological development (e.g., Chrousos et al., 1998; Spear, 2000; Steinberg, 2005).

Increasing age and puberty affect HPA axis maturation reflected by a general increase in basal levels of cortisol (Gunnar et al., 2009) and a flatter diurnal slope (e.g., Shirtcliff et al., 2012). The neurobiological change during early adolescence is likely associated with an increase in environmental demands (e.g., social; Blakemore & Mills, 2014; Crone & Dahl, 2012) and vulnerability for the onset of psychiatric conditions (e.g., depression; Breslau et al., 2017; Gunnar et al., 2009; Kessler et al., 2005). In fact, evening cortisol has been associated with higher rates of depression in adolescents 13–18 years (Van den Bergh & Van Calster, 2009).

Furthermore, like many aspects of maturation, the magnitude and shape of the slope is different for males and females. Sex-based comparisons reveal that across adolescence, females evidence a stronger circadian rhythm as well as higher basal cortisol (Rosmalen et al., 2005; Shirtcliff et al., 2012), which are primarily driven by age rather than earlier entry into puberty in females (Shirtcliff et al., 2012). It has been speculated that such differences in HPA profiles may be related to differential emergence of various mental and physical health conditions between females and males (Zahn-Waxler et al., 2008).

In addition to developmental factors, Dallman et al. (2004) highlight that HPA axis regulation is influenced by metabolic factors such as body mass index (BMI) (e.g., Dallman et al., 2004; Rosmalen et al., 2005; Shirtcliff et al., 2012). Also, certain medications have been shown to impact HPA axis activity (see Granger et al., 2009). As a result, BMI and medication factors must be considered when examining diurnal cortisol measurement and analysis.

### Autism spectrum disorder

The integrity of the HPA axis has been explored in autism spectrum disorder (ASD), a neurodevelopmental disorder defined by impairments in reciprocal social communication and restricted and repetitive interests and behaviors (APA, 2013). Notably, autistic children have heightened sensory sensitivity (e.g., APA, 2013; Tomchek & Dunn, 2007) and poor adaptability to change (Walker et al., 2001) which can lead to increased stress and arousal. ASD occurs more often in males than females ranging from a 4:1 male-to-female ratio (Maenner et al., 2023) to 2:1 or 3:1 with such discrepancies ostensibly due to under-diagnosis (Kim et al., 2011; Loomes et al., 2017) and a unique female phenotype (Corbett et al., 2020; Kreiser & White, 2014; Mandy et al., 2012; Uljarevic et al., 2020). Currently, there is debate regarding the use of terminology and whether person-first language in which the individual (e.g., adolescent) is referenced before the condition (e.g., autism) or whether identity-first language (e.g., autistic adolescent) should be used. When formally referencing the diagnostic or group category, the authors have opted to use the term ASD; otherwise, the term autistic will be used.

Elevation in cortisol during the pubertal transition is adaptive and helps prepare youth for the increased demands and novel challenges characteristic of adolescence. However, adolescence is postulated to be a time of increased susceptibility in autistic youth (Picci & Scherf, 2015), in part due to enhanced physiological arousal (Muscatello & Corbett, 2018) and poor adaption to change (Walker et al., 2001) including developmental transitions (Taylor et al., 2017; Taylor & Seltzer, 2010). Investigations examining the integrity of the HPA axis in autistic children have shown more variable and dysregulated rhythms (Corbett et al., 2006; Corbett et al., 2008; Corbett et al., 2009; Hoshino et al., 1987; Tomarken et al., 2015). Also, elevated evening cortisol has been consistently found in autistic children and adolescents compared to TD peers (Corbett et al., 2009; Tordjman et al., 2014), which contributes to a blunted slope in at least a subgroup of children (Tomarken et al., 2015). However, previous research on the CAR does not distinguish autistic children from their TD peers (Corbett & Schupp, 2014; Taylor & Corbett, 2014; Zinke et al., 2010). Although differences have been reported in one study of autistic adolescents (Brosnan et al., 2009), the CAR was reported to be associated with self-report of depression in autistic females (Sharpley et al., 2016).

While not the focus of the current study, it is important to note that enhanced social stress as measured by cortisol has been frequently reported (Corbett et al., 2009, 2012; Corbett et al., 2010) with increased cortisol responsivity with age (Schupp et al., 2013) and pubertal development (Muscatello & Corbett, 2018). Amidst the differences demonstrated between autistic females and males such as pubertal timing (Corbett et al., 2020), internalizing symptoms (Hedley et al., 2018; May et al., 2014; Schwartzman et al., 2022), and social communication (Wood-Downie et al., 2021), previous research interrogating the HPA axis has been limited by relatively few or absent female participants (Corbett & Schupp, 2014; Edmiston et al., 2017).

To date, research examining the diurnal regulation of cortisol has shown atypical patterns postulated to be related to heightened sensitivity to change, events of the day and sensory sensitivity (e.g., Corbett et al., 2009). What is less clear is the extent to which such predisposition is further impacted by physiological and environmental changes brought on by puberty and adolescence, respectively.

The purpose of the current study was to extend previous research in diurnal cortisol (i.e., slope, CAR, evening) by (1) studying cortisol expression longitudinally over the pubertal transition between autistic and neurotypical youth, (2) determine the trajectory of diurnal cortisol (collected over 3 consecutive weekdays) and the unique contributions of age vs. puberty, and (3) examine potential sex differences in female and male youth. The following hypotheses (Hyp) are advanced: Hyp 1. Circadian Rhythmicity (slope, CAR and evening): it is hypothesized that autistic youth compared to TD youth will have a shallower slope, higher evening cortisol and no group differences based on the CAR; Hyp 2. Impact of Age and Puberty: it is hypothesized that as the youth develop, they will have higher cortisol and flatter circadian rhythms based on age (Hyp 2.1) and pubertal development (Hyp 2.2); and Hyp 3. Sex-Based Differences: It is hypothesized that females in both groups (ASD/TD) will have higher cortisol, flatter slopes, and higher evening cortisol than males. Additionally, females with higher evening cortisol will have more depression symptoms.

## Method

The research was carried out in accordance with the Code of Ethics of the World Medical Association (Declaration of Helsinki). The Vanderbilt Institutional Review Board approved the study. Informed written consent and assent was obtained from all parents and study participants, respectively, prior to inclusion in the study.

### Participants

Data were collected as part of a longitudinal study on pubertal development and stress (Corbett, 2017). The current study includes data from the first three assessment years: Year-1 (Y1) enrollment when the children were between 10-years-0-months to 13-years-11-months of age, Year-2 (Y2) 1 year after the participant’s initial visit, and Year-3 (Y3) 1 year after Y2. Diagnostic procedures were completed in Y1. The physical exam (Tanner staging), physiological measures (e.g., cortisol), and psychological testing and questionnaires (e.g., CBCL) were completed annually.

In Y1, the sample included 245 total youth, with 239 participants that completed the physical exam described below. The ASD group consisted of 140 participants (median age 11.2) including 36 females and 104 males. The TD group consisted of 105 participants (median age 11.7) including 46 females and 59 males. One autistic male was missing a measurement for G stage, and one TD female was missing measurement for PH stage.

For Year 1, the racial and ethnic characterization of the sample was comprised of 7.8% Black, 83.3% White, and 8.6% multiracial. Participants were recruited from a broad community sample in the southern United States covering a 200-mile radius that targeted medical and health-related services, clinics, research registries, regional disability organizations, schools, and social media platforms. Inclusion required an IQ score ≥ 70 due to task demands in the source longitudinal study. Children were excluded if taking medications that alter the Hypothalamic-Pituitary-Adrenal (HPA) axis (e.g., corticosteroids; see Granger et al., 2009) or HPG axis (e.g., growth hormone), or medical condition known to impact pubertal development (e.g., Cushing’s Disease). Demographic information for each group is presented in Table 1.

In Y2 there were 174 participants, the ASD group had a median age of 12.5 years, and the TD group had a median age of 12.7 years. The overall attrition rate was 26.89%, which was comparable to other longitudinal studies after the initial enrollment (Negriff et al., 2015). At Y3, there were 163 participants, with a median age of 13.3 years for the ASD group and 13.8 years for the TD group. At Y2 and Y3, some participants were unable to complete the full physical examination due to restrictions on in-person lab visits resulting from the COVID-19 pandemic (Y2 *N* = 43; Y3 *N* = 59).

### Diagnostic procedures and assessment measures

The diagnosis of ASD was based on the Diagnostic and Statistical Manual-5 (APA, 2013) and confirmed by an established diagnosis by a psychologist, psychiatrist, or behavioral pediatrician with ASD expertise, current clinical judgment by a study team member, and corroborated by the Autism Diagnostic Observation Schedule (ADOS-2; Lord et al., 2012).

#### Autism Diagnostic Observation Schedule-Second Edition

(ADOS-2; Lord et al., 2012) is a semi-structured interactive play and interview-based instrument used to support the diagnosis of ASD. The ADOS Module III was administered by research-reliable personnel.

#### Social Communication Questionnaire

(SCQ; Rutter et al., 2003) is a screening questionnaire to assess for symptoms of ASD. A score of 15 is suggestive of a diagnosis of ASD. Due to lower sensitivity and specificity (Barnard-Brak et al., 2016), TD children with a score ≥ 10 were excluded from the study.

#### Wechsler Abbreviated Scale of Intelligence, Second Edition

(WASI-II, Wechsler, 2011) a measure of cognitive ability, was used to obtain an estimate of the participant’s intellectual functioning. Inclusion for the study required an IQ ≥ 70.

#### The Child Behavior Checklist

(CBCL; Achenbach & Rescorla, 2001) is a broad-based parent report form used to provide children’s competencies and behavioral/emotional problems from 6 to 18 years of age. The CBCL affective (Depression) domain was used in regression data analysis. The psychometric properties of the CBCL are good. Specifically, the test retest intraclass correlation for the DSM-Oriented scales is *r* = 0.88 and the internal consistency alpha ranges from 0.72 to 0.91.

### Physical exam

The standardized physical exam was completed in a clinical exam room during each annual visit to reliably identify pubertal development and assign Tanner stage (Marshall & Tanner, 1969, 1970). The exam ascertained two measures with 5 stages for Male External Genitalia (G1–G5 for males) and Female Breast (B1–B5 for females) (G/B stage) and Pubic hair (P1–P5 for both sexes) (PH stage). The exam consisted of visual inspection and categorization of pubertal and genital maturation. To be consistent with the original Tanner staging and to maximize participation, palpation of breasts or measurement of testes was not conducted.

The exam was conducted by fully trained, licensed study physicians. A male physician conducted most of the exams; however, a female physician provided same-sex exams as requested. Previous research has shown that the sex of the physician is not as important as the participant’s comfort level and the competence of the physician (Dorn et al., 2006). To this end, the physicians first established rapport, explained the rationale for the exam and addressed any questions or concerns. Next, the participant’s height and weight were measured. Subsequently, the adolescent was requested to loosen clothing to fully expose breast and lower genital region, rather than fully disrobing. This approach aided in the level of comfort for the participants. A companion (e.g., parent) was offered to accompany the participant in the exam room.

Physician inter-rater reliability was established between study physicians on 10 randomly selected participants. Cohen’s Kappa (κ) was calculated between study physicians to assess the degree to which raters were able to identify Tanner stages for G/B and PH markers. Inter-rater reliability for markers ranged from κ= 0.62 to 0.75 (all *p* < 0.001; substantial agreement). Absolute agreement was 0.75. Kappa was also calculated to assess the extent to which physicians were able to reliably and independently identify when participants had initiated pubertal maturation (Stage 2) for each marker. Kappa ranged from 0.62 to 1.00 (good to very good). In cases of disagreement, physician ratings were never greater than one stage difference.

### Cortisol sampling procedure

Diurnal cortisol saliva samples were collected at home four times a day: In the morning following natural waking (Edwards et al., 2001; Kudielka & Kirschbaum, 2003; Wilhelm et al., 2007), 30-min Post Waking, Afternoon, and Evening, over 3 consecutive weekdays (non-holidays) immediately prior to the in-lab visit using established procedures (e.g., passive drool, postponed if sick; Corbett et al., 2006, 2008). Families and participants were rigorously trained on collection procedures, received instructional materials and DVD demonstrations, and were prompted with sampling reminders in the days prior to and on scheduled sampling dates. Furthermore, families completed daily diaries to track sample times, in addition to recording the collection time on sample labels. Diaries also included prompts for recording time to bed, time woken, total sleep, and any important notes about the day (e.g., early release from school, atypical schedule). For two children who had difficulty with the passive drool method because of oral motor or sensory aversion issues, a cotton roll and syringe procedure was used for each of their samples (Tordjman et al., 2014). Sensitivity analyses were conducted excluding these two children and results were not meaningfully different, and we proceed with the full dataset. Participants were instructed to not eat or drink 1-hr prior to salivary collection and to refrain from brushing teeth in the morning until after Post Waking. Per the protocol, participants passively drooled into a test tube using a straw collecting approximately 1 mL of saliva. Participants were instructed to collect home salivary samples in the 3 weekdays prior to the lab visit. Samples were refrigerated in the home until returning to the lab at which time they were placed in a −80°C freezer. In an effort to account for hormonal changes throughout the menstrual cycle, female participants that had begun menstruating were scheduled during the luteal phase, based on date of last menses, as previous research has shown women in the luteal phase to have comparable cortisol levels to men (Kirschbaum et al., 1999).

### Cortisol assay

Cortisol assays were performed using a Coat-A-Count^®^ radioimmunoassay kit (Siemens Medical Solutions Diagnostics, Los Angeles, CA) modified to accommodate lower levels of cortisol in human saliva. Samples stored at –80°C, were thawed and centrifuged at 3460 r.p.m. for 15 min to separate the aqueous component from mucins and other suspended particles. The coated tube from the kit was substituted with a glass tube into which 100 ul of saliva, 100 ul of cortisol antibody (courtesy of Wendell Nicholson, Vanderbilt University, Nashville, TN), and 100 ul of ^125^I-cortisol were mixed. After incubation at 4°C for 24 hr 100 ul of normal rat serum in 0.1% PO4/EDTA buffer (1:50) and precipitating reagent (PR81) were added. The mixture was centrifuged at 3460 r.p.m. for 30 min, decanted, and counted [for details see Corbett et al. (2013)]. Serial dilution of samples indicated a linearity of 0.99. The intra-assay coefficients of variation were as follows Y1 CV = 2.06%; Y2 CV = 2.80%; Y3 CV = 1.90%. The total intra-assay (across all 3 years) = 2.22%. The inter-assay CV was 9.52%.

### Aims, hypotheses and definitions

#### 1. Circadian Rhythmicity:

The aim was to examine diurnal cortisol longitudinally (slope, CAR, and evening) between the groups (ASD & TD). Slope was comprised of 4 samples: Waking, Post Waking, Afternoon, and Evening for 3 consecutive days. For analyses, each of these cortisol time periods was modeled across the 3 sampling days across 3 years with random effects for hypothesis 1, 2, and 3.1, but evening values were averaged for hypothesis 3.2. The CAR was calculated by subtracting the 30-min sample from immediate morning sample across the three days. Hyp1.1: It was hypothesized that autistic youth compared to TD youth will have a more shallow slope, higher evening cortisol and no group differences based on the CAR. Hyp 1.2: It was hypothesized that autistic youth will have elevated evening cortisol.

#### 2. Developmental Trajectory:

The aim was to examine diurnal slope in consideration of age and pubertal development separately and together. Puberty was examined by physical exam alone (without age in the model) as a marker of development. Age and Puberty were also examined together to evaluate the possibility of combinatory effect on cortisol levels. Hyp 2.1: It was hypothesized that as the youth age, they will have higher cortisol and flatter circadian rhythms. Hyp 2.2: It was hypothesized that cortisol levels will be higher as the groups advance through puberty. Moreover, the slopes will become flatter. Hyp 2.3: It was hypothesized that the effect of puberty will be less or nonsignificant when controlling for age.

#### 3. Sex-Based Differences:

The aim was to examine diurnal slope and evening cortisol over development based on sex. Sex was defined as biological sex assigned at birth to compare sex (males/females) while controlling for key factors (BMI and medication use). Hyp 3.1: It was hypothesized that females in both groups (ASD/TD) will have higher cortisol, flatter slopes, and higher evening cortisol than males. *Depression associations:* The CBCL Affective Problems index was used to determine if sex differences exist by looking at associations between sex (females) and depression. Since CBCL scores were only collected yearly, evening cortisol was taken to be the average of the three evening samples for this analysis. Hyp 3.2: It was hypothesized that females with higher evening cortisol will have increased depression symptoms.

### Statistical analyses

Characteristics of participants were stratified by diagnosis and summarized in Table 1, using proportions and frequencies for categorical measures, and the 1st, 2nd (median), and 3rd quartiles for numerical measures. Cortisol values were positively skewed; therefore, values were log10 transformed prior to statistical analyses. Additionally for categorical variables, Pearson’s χ2 tests of homogeneity were conducted to identify whether the frequency distributions differed between the two groups (TD vs. ASD). Finally, Wilcoxon rank-sum tests were conducted for continuous variables to test whether the probability distributions differed by diagnosis. The null hypothesis in each test purported that there was no difference in distributions between the two groups.

To test Aim 1, we fit a linear mixed effects model on log10 transformed cortisol values. We modeled diurnal cortisol levels including diagnosis by time period (period) and age by period interactions, controlling for sex, body mass index (BMI), and whether the patient was on medication. To account for the correlation structure, random intercepts for subject, year, and day (ordered as nested) were included in the model. For this and all subsequent models, we used heteroskedasticity consistent standard errors and performed tests using type 2 sum of squares analysis of variance. To test whether the slope differs between groups at the 3 period transitions of interest, – CAR (Post Waking – Waking), Afternoon – Post Waking, & Evening – Post Waking, – we calculated 2-way pairwise contrasts between periods and diagnosis (ASD-TD) while controlling for the period by age interaction. In all models, when performing multiple tests across timepoints we used the Bonferroni correction to adjust for the three multiple comparisons. To test whether the ASD group has a higher evening cortisol level than the TD group (Hyp 1.2), we conducted a linear contrast of the evening cortisol levels between TD and ASD youth.

To investigate hypothesis 2.1, we first modeled and tested log10 diurnal cortisol levels including a diagnosis by period by age interaction, while controlling for sex, body mass index (BMI), and whether the participant was on medication. After, we considered a model without the three-way interaction and including only the diagnosis by period and age by period interaction (as in Hyp 1). For hypothesis 2.2, we considered the above models with a pubertal development effect in lieu of age. In all these models, random intercepts for subject, year, and day (nested as ordered) were included to account for the correlation structure in the data. We considered nonlinear effects for age and puberty using natural cubic splines but retained linear effects only after comparing model fit with AIC. To test if age increases cortisol levels, we tested the main effect of age using the hypothesis 2.1 model excluding the age–period interaction with type II ANOVA. To test whether age has a flattening effect on circadian cortisol responses throughout the day, we conducted period contrasts of the age trends. These testing procedures were mirrored for hypothesis 2.2, replacing age with pubertal stage. To test if the effect of puberty exists after controlling for age (Hyp 2.3), we fit a model including both the age by period and pubertal stage by period interactions as well as a model with an age by period by pubertal stage interaction.

Additionally, we considered a separate random effects structure adding a random intercept for period nested within subject. These models also allowed the day and period random effects variability to vary between diagnosis groups. Ultimately, the random effects variance did not significantly differ between the groups (Table S1), and the simpler random effects structure was retained for all analyses.

To test hypothesis 3.1, we expanded the linear mixed effects model from hypothesis 2.1 to include a sex by period interaction term. To test whether females have higher cortisol, we tested the main effect of sex in the linear mixed effects model fit in hypothesis 2.1 (no sex-period interaction). To test whether females have shallower slopes in regard to cortisol changes throughout the day, we conducted sex contrasts of the slope between each time period contrast. We tested whether females had higher evening cortisol levels than males using a linear contrast of the evening cortisol levels by sex. Next, to address hypothesis 3.2, using only data on the 69 females, we considered a mixed effects model on affective depression scores including evening mean cortisol (across year) as the variable of interest while controlling for BMI and existence of medication. This second model only included random intercepts for subject because depressive scores were measured on a year-toyear basis. All analyses were performed using R 4.2.1.

## Results

### Hyp 1.1:

**Figure 1.**
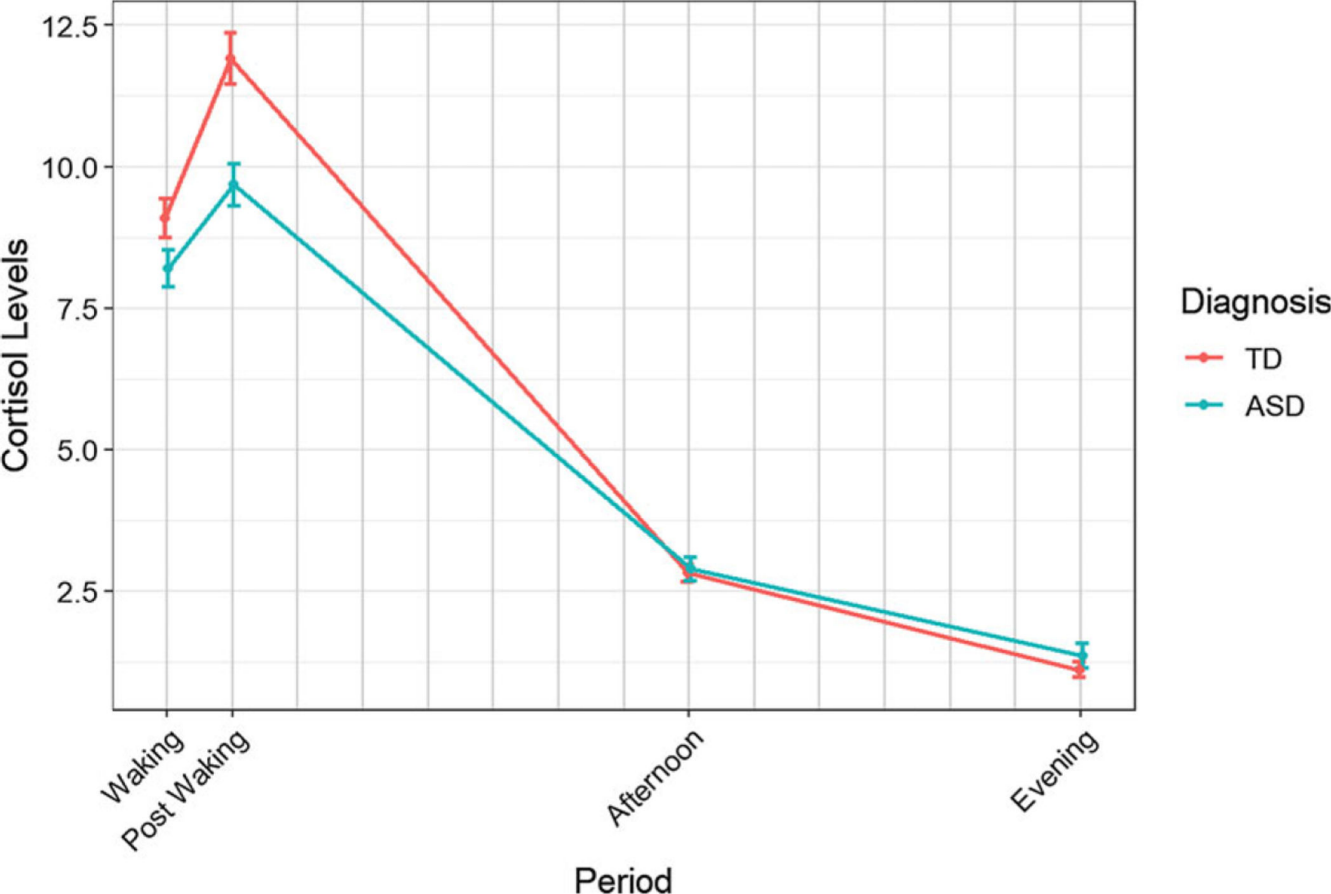
Mean diurnal cortisol levels over the course of the day. Note. The figures depict raw untransformed or log-transformed diurnal cortisol levels, which unlike the model, do not adjust for covariates nor the within subject correlation

To investigate differences in mean cortisol and diurnal cortisol response between groups we fit a linear mixed effects model and tested components of the diagnosis by period interaction. There was not sufficient evidence to detect differences between the groups for the CAR Post Waking – Waking response (corrected *p* = 0.3406; Table 2). There was evidence of a difference between the groups in terms of the Afternoon – Post Waking (corrected *p* = 0.0001; Table 2). The contrast estimate (0.1113) shows that the ASD group has a shallower cortisol slope over this period (Figure 1). The expected baseline cortisol change from Post Waking to Afternoon is an approximate 77.50% decrease in cortisol levels. Over this transition, for ASD, the expected decrease in cortisol levels is 29.21% less extreme than TD. The coefficient table, random effects estimates, and ANOVA tables are given in the Supplementary material (Table S2, S3).

We also saw a significant difference between the groups in terms of the Evening – Post Waking (corrected *p* < 0.0001; Table 2). The contrast estimate (0.1803) shows that the ASD group has a shallower cortisol slope over this period transition. The expected baseline cortisol change from Post Waking to Evening is an approximate 93.81% decrease in cortisol levels. For autistic youth, the expected decrease in cortisol levels is 51.48% less extreme than TD youth.

### Hyp 1.2:

Within the same model, we tested the difference in evening cortisol levels between TD and ASD. This contrast shows that autistic youth have a significantly higher cortisol level in the Evening (*p* = 0.0117) when compared to TD youth (Table 2). On average in the evening, youth with ASD have 25.01% higher cortisol levels when compared to TD youth.

### Hyp 2.1:

**Figure 2.**
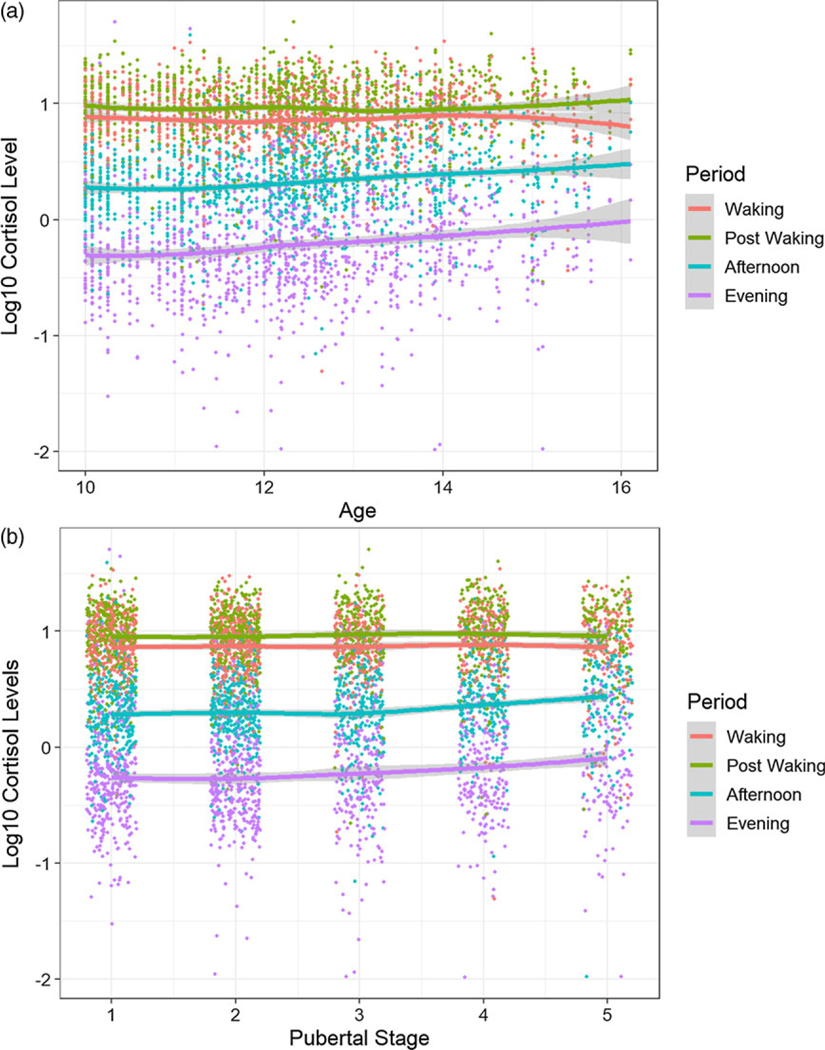
(a) Observed log cortisol vs. age with smoothing (loess) curve for all 4 periods. (b) Observed log cortisol vs. pubertal stage with a smoothing (loess) curve for all 4 periods.

To investigate the effect of age on the whole diurnal cortisol trajectory, we tested the main effect of age without the age–period interaction, which showed cortisol levels increase 5.78% with each year aged (Table 3). To investigate the effect of age on the shape of the diurnal cortisol trajectory we tested interaction of age with specific components of the trajectory. There was not sufficient evidence for a difference in age trends for the CAR (*p* = 1.000; Table 3). However, we did find evidence for a difference in age trends for the Afternoon – Post Waking change (corrected *p* < 0.0001) indicating that age has a flattening effect on the cortisol slope over this period (Table 3; Figure 2a). Specifically, for each year aged, there is a 9.14% decrease in the change of cortisol levels between Post Waking and Afternoon measurements. We also find a flattening effect in the Evening – Post Waking change (corrected *p* = 0.0004; Table 3). Specifically, the expected decrease in cortisol levels over this period transition is 12.65% less extreme for each year aged.

### Hyp 2.2:

To investigate the effect of puberty on the whole diurnal cortisol trajectory, we tested the main effect for pubertal stage shown to have significant positive effect on cortisol levels (Table 4). We found that for each stage advanced through puberty, the expected cortisol level would rise by 5.52%.

To investigate the effect of puberty on the shape of the diurnal cortisol trajectory, we tested interaction of pubertal stage with specific components of the trajectory. There was not sufficient evidence for a difference in pubertal stage trends for the CAR (Post Waking – Waking) and Evening – Post Waking responses (Table 4). However, there was evidence for a difference (corrected *p* = 0.0149) in puberty stage trends for the Afternoon – Post Waking transition which indicated that puberty stage has a flattening effect on the cortisol slope over this transition (Table 4). For each pubertal stage advanced, the expected decrease in cortisol levels over this transition is 6.93% less extreme (Figure 2b). The coefficient table, random effects estimates, and ANOVA tables are given in the Supplementary material (Table S4, S5).

### Hyp 2.3:

After including the age by period interaction in the model, the interaction between pubertal stage and period became less significant (Table S7). Further, none of its levels are individually significant in the model after the age–period interaction inclusion (Table S6). Additionally, the three-way interaction between age, puberty, and period was not significant.

### Hyp 3.1.

**Figure 3.**
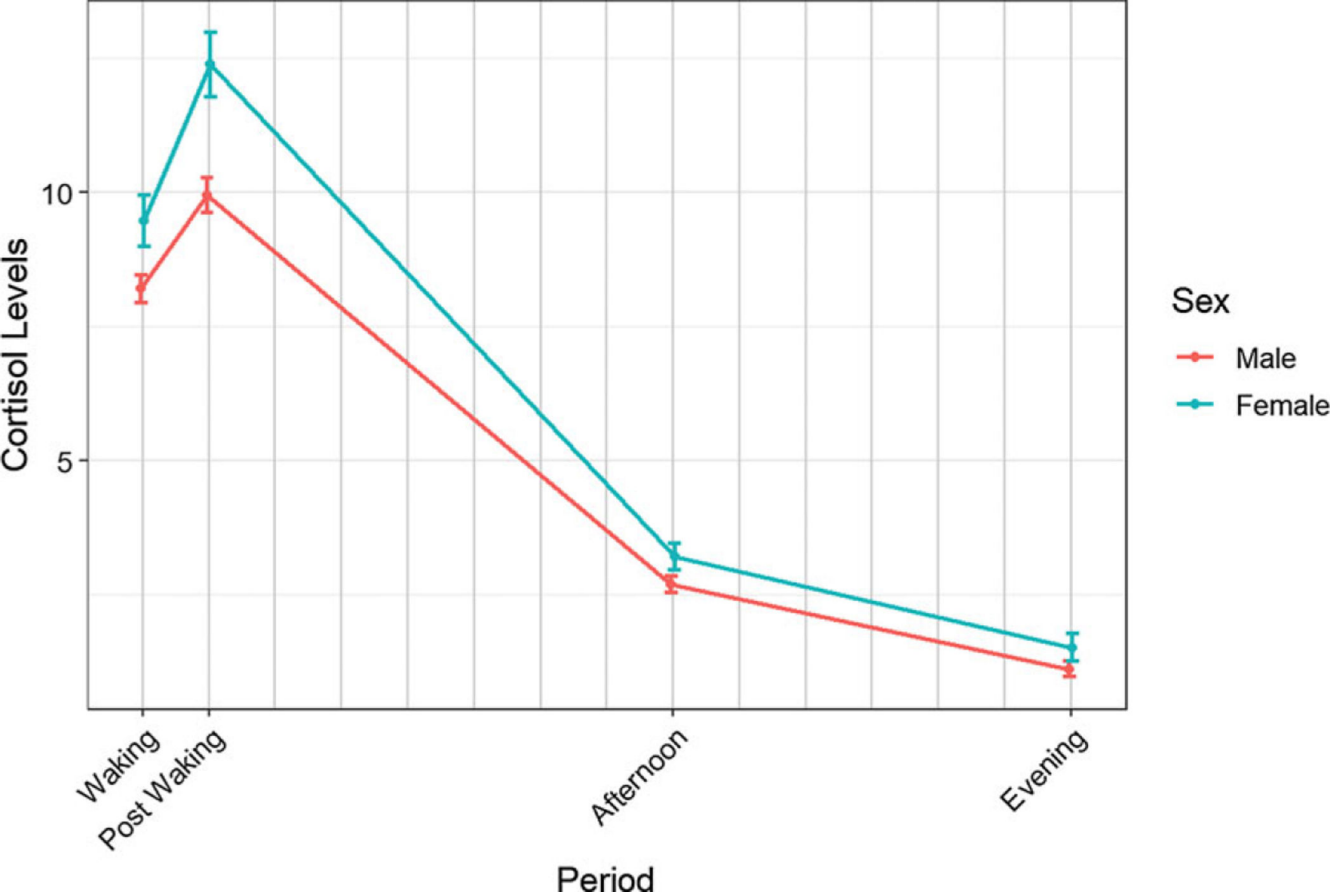
Mean diurnal cortisol levels over the course of the day stratified by sex. Note. The figures depict raw untransformed or log-transformed diurnal cortisol levels, which unlike the model, do not adjust for covariates nor the within subject correlation.

Testing the main effect for sex showed females have 22.10% higher cortisol levels (Table 5; Figure 3). There was no statistically significant difference between the sexes for the CAR, Afternoon – Post Waking, and Evening – Post Waking responses (Table 5). Testing the sex difference in evening cortisol levels showed that females have significantly (*p* < 0.0001) higher cortisol than males on average, with females expected to have 50.19% higher cortisol levels in the evening compared to males. The coefficient table, random effects estimates, and ANOVA tables are given in the Supplementary material (Table S8, S9).

### Hyp 3.2:

We did not find sufficient evidence for an association between Evening mean cortisol and depressive symptoms in females (B = 0.234, 95% CI =−0.486, 0.953, *p* = 0.519; Table S10).

## Discussion

The purpose of the study was to extend previous research in diurnal cortisol rhythmicity by (1) studying cortisol expression longitudinally over the pubertal transition between autistic and neurotypical youth, (2) determine the trajectory of diurnal cortisol and the unique contributions of age vs. puberty, and (3) examine potential sex differences in female and male youth. As predicted, autistic youth compared to TD youth demonstrated a shallower slope reflected by significant differences between the Post-waking and Afternoon and the Post Waking and Evening cortisol values. Amidst the expected 77.50% and 93.81% decrease in slope, between Post Waking and Afternoon and Evening cortisol, autistic youth showed a reduced slope of 29.21% and 51.48% in the afternoon and evening, respectively. The findings of a reduced slope replicate previous research in diurnal cortisol in ASD (Tomarken et al., 2015) yet with a much larger sample of children and adolescents broadly ranging between 10 and 16 years of age. The shallow slope is also consistent with research directly comparing a blended sample of autistic and TD children vs. adolescents aged 7–17 years showing a blunted diurnal slope attributed to group differences in morning and evening cortisol (Muscatello & Corbett, 2018).

The direct period contrasts revealed higher evening cortisol in autistic youth compared to TD youth, a finding that has been reported across several cross-sectional studies (Corbett et al., 2006, 2008, 2009; Muscatello & Corbett, 2018; Tomarken et al., 2015). In the current prospective study, evening cortisol in autistic youth was 25.01% higher when compared to TD youth. In ASD, elevated evening cortisol has been associated with cumulative daily stress throughout the day (Corbett et al., 2009), which may reflect persistent heightened arousal contributing to a hyperactive HPA axis (Richdale & Prior, 1992). Autistic children often have poor response to change (Walker et al., 2001), a pattern that has been explicitly linked to evening cortisol (Corbett et al., 2009). Thus, cumulative stress as a result of changing environmental demands contribute to increased arousal late in the day reflected in elevated cortisol in autistic youth. Higher arousal levels have been linked with sleep problems in autistic children (Richdale et al., 2014). However, due to the lack of difference in CAR in the current sample, sleep dysregulation appears a less likely factor related to the elevated cortisol in the evening. In non-ASD samples, elevated HPA activity and atypical sleep patterns have been characterized as risk markers for the onset of depression in high-risk adolescents (Rao et al., 2009). In youth, elevated evening cortisol has been associated with depression and linked to a variety of physical and mental health conditions (Lupien et al., 2009), which warrant greater study.

Taken together, it is apparent that autistic youth evidence differences in the diurnal slope and cortisol levels in the evening. The results are consonant with previous research and strongly point to greater responsivity to external (e.g., poor adaptation to change) and internal (e.g., increased sensory sensitivity) factors in autistic youth resulting in cumulative stress manifest as elevations in cortisol.

Subsequently, we examined the developmental trajectory of the diurnal slope in consideration of age and pubertal development separately and together. The results supported the notion that age has a flattening effect on the diurnal cortisol slope from Post Waking to Afternoon as well as Post Waking to Evening. When examining age trends across diurnal periods, the slope from Post waking to Afternoon decreased with advancing age (see Figure 2a). Muscatello and Corbett (2018) reported that in addition to group differences, older autistic adolescents showed a flatter slope due to higher evening cortisol and lower morning values. It appears that in addition to the adaptive rise in basal cortisol and flatter diurnal slope (Gunnar et al., 2009; Shirtcliff et al., 2012), autistic youth evidence even higher basal cortisol during adolescence. In other words, both diagnostic and developmental effects contribute to higher cortisol in autistic youth.

Similar to age effects, significant difference in pubertal stage trends was observed for the Afternoon – Post Waking diurnal period resulting in a flattening of the slope. When pubertal trends were examined across diurnal periods, the slope from Post waking to Afternoon grew more shallow with advancing pubertal development (see Figure 2b). These results confirm hypotheses and are consistent with the limited cross-sectional research in ASD (e.g., Muscatello & Corbett, 2018).

As predicted, there were no differences between autistic and TD youth based on the CAR. The finding is consistent with the extant literature (Corbett & Schupp, 2014; Taylor & Corbett, 2014; Zinke et al., 2010) with the exception of one study of adolescents with ASD (Brosnan et al., 2009).

Since many previous studies in autism have been conducted with male youth only or very few females, the current study made a significant contribution by including a relatively large group of autistic females. Sex-based differences were explored for diurnal slope and evening cortisol over development predicting that females in both groups would have higher cortisol, flatter slopes, and higher evening cortisol than males. The hypothesis was partially confirmed showing that females have higher evening cortisol levels than males such that females have a 50.19% higher cortisol level when compared to males. Studies in typically developing youth have revealed higher basal cortisol and stronger circadian rhythm in females driven primarily by age rather than earlier pubertal development (Rosmalen et al., 2005; Shirtcliff et al., 2012). Moreover, it has been surmised that sex-based differences in HPA profiles may be related to differential emergence of various mental and physical health conditions between males and females (Zahn-Waxler et al., 2008).

Based on results showing elevations in cortisol and associations with depression in neurotypical (e.g., Van den Bergh & Van Calster, 2009) and autistic female (e.g., Sharpley et al., 2016) adolescents, these associations were explored. Surprisingly, evening cortisol did not predict symptoms of depression in autistic females in the current study. Thus, despite being at higher risk for depression (Schwartzman et al., 2022), elevated evening cortisol (Corbett et al., 2006, 2008; Corbett et al., 2009; Muscatello & Corbett, 2018; Tomarken et al., 2015), and advanced puberty (Corbett et al., 2020), there was no observed association between cortisol level and symptoms of depression. It is unknown if a connection would exist in autistic females with a confirmed diagnosis of depression. A recent meta-analysis reported that elevated morning and nocturnal cortisol may be risk factors for onset of major depressive disorder (MDD) in adolescence or young adulthood (Zajkowska et al., 2022) such that hyperactivity of the HPA precedes and contributes to the onset of depression during adolescence. The current study did not characterize the sample with regard to confirming the co-occurrence of MDD, rather a symptom profile was used. Nevertheless, symptoms of depression are more prevalent in ASD during early adolescence especially in females (Schwartzman et al., 2022). Thus, it remains a cautionary tale and expanded research is needed to explore potential links between elevated cortisol as a risk factor for developing depression in autistic youth especially females.

### Strengths, limitations, and future directions

Although previous research has examined the integrity of the HPA axis in ASD, the research has been largely cross-sectional, with small-to-moderate sample sizes, a lack of female participants, and use of a questionnaire to determine pubertal status (e.g., Corbett et al., 2006, 2008; Edmiston et al., 2017; Muscatello & Corbett, 2018; Tordjman et al., 2014). The current study addresses these gaps by utilizing a 3-year prospective longitudinal design, inclusion of a large, well-characterized sample including autistic females, and use of gold-standard pubertal Tanner staging. Even so, limitations remain to include a lack of participants with intellectual disability and less representation of minoritized individuals. Further, during Y2 and Y3, some participants were unable to complete the full physical examination due to restrictions on in-person lab visits resulting from the COVID-19 pandemic (Y2 *N* = 43; Y3 *N* = 59), which only affected models with pubertal stage as a variable. Additionally, to measure symptoms of depression, only a subdomain from a broad parent-report measure was used rather than a focused psychiatric interview. Future studies are needed to be more inclusive in terms of race, ethnicity, SES, and diagnostic severity. Furthermore, to more rigorously measure co-occurring conditions (e.g., depression), a standardized psychiatric interview may be optimal to capture the nuance of mental health risk profiles. Lastly, while menstruating females were scheduled during the luteal phase of their cycle, future research with larger samples of females will benefit from rigorous menstrual cycle tracking (e.g., Backward and Forward counting method). Similarly, tracking and controlling for other environmental factors that may influence the HPA axis, such as seasonality, will be important.

In summary, the current study extended research in ASD during the pivotal adolescent and pubertal transition and revealed that despite the trait-like stability of diurnal cortisol, HPA maturation is impacted by age, puberty, sex as well as an ASD diagnosis. Questions remain as to the long-term medical sequelae and mental health outcomes in autistic youth from dysregulated HPA axis. Further, an enhanced understanding of moderating effects on the differences in diurnal slope and basal levels will inform developmental risk during adolescence and the pubertal transition to adulthood.

## Supplementary Material

1

## Figures and Tables

**Table 1.** Descriptive statistics stratified by diagnosis

		TD	ASD	Overall	
	*N*	(*N* = 105)	(*N* = 140)	(*N* = 245)	Test statistic

Sex: female	245	0.44 46/105	0.26 36/140	0.33 82/245	X^2^(1) = 8.82^2^**

Ethnicity: Hispanic	245	0.05 5/105	0.08 11/140	0.07 16/245	X^2^(1) = 0.94^2^

Age	245	10.58 **11.67** 12.67	10.50 **11.25** 12.25	10.58 **11.33** 12.36	F_1,243_ = 3.06^3^

Race					

Caucasian	245	0.86 90/105	0.81 114/140	0.83 204/245	X^2^(3) = 12.10^2^**
		
African American		0.02 2/105	0.12 17/140	0.08 19/245	
		
American Indian		0.00 0/105	0.00 0/140	0.00 0/245	
		
Asian or Pacific Islander		0.00 0/105	0.01 1/140	0.00 1/245	
		
More than one		0.12 13/105	0.06 8/140	0.09 21/245	

G/B Development Change	105	1.00 **1.00** 2.00	1.00 **1.00** 2.00	1.00 **1.00** 2.00	F_1,103_ = 0.02^3^

Pubic Development Change	106	1.00 **1.50** 2.00	1.00 **2.00** 2.08	1.00 **2.00** 2.00	F_1,104_ = 1.16^3^

Mean of Log Waking Cortisol Year 1	210	0.82 **0.92** 1.04	0.75 **0.88** 1.01	0.77 **0.90** 1.02	F_1,208_ = 3.28^3^

Mean of Log Post Waking Cortisol Year 1	208	0.92 **1.04** 1.13	0.81 **0.97** 1.09	0.86 **1.00** 1.11	F_1,206_ = 6.84^3^**

Mean of Log Afternoon Cortisol Year 1	209	0.18 **0.29** 0.44	0.19 **0.33** 0.46	0.19 **0.31** 0.44	F_1,207_ = 1.27^3^

Mean of Log Evening Cortisol Year 1	209	−0.53 −**0.35** −0.12	−0.41 −**0.23** −0.00	−0.48 −**0.32** −0.06	F_1,207_ = 5.57^3^*

Mean of Log Waking Cortisol Year 2	172	0.77 **0.88** 0.98	0.67 **0.85** 0.97	0.72 **0.88** 0.97	F_1,170_ = 2.96^3^

Mean of Log Post Waking Cortisol Year 2	170	0.82 **0.98** 1.14	0.77 **0.94** 1.05	0.82 **0.96** 1.09	F_1,168_ = 3.41^3^

Mean of Log Afternoon Cortisol Year 2	171	0.22 **0.31** 0.45	0.12 **0.28** 0.45	0.15 **0.30** 0.45	F_1,169_ = 1.18^3^

Mean of Log Evening Cortisol Year 2	170	−0.45 −**0.27** −0.04	−0.47 −**0.22** 0.03	−0.47 −**0.25** 0.01	F_1,168_ = 0.96^3^

Mean of Log Waking Cortisol Year 3	157	0.81 **0.91** 1.02	0.69 **0.84** 0.95	0.75 **0.89** 0.96	F_1,155_ = 5.91^3^*

Mean of Log Post Waking Cortisol Year 3	154	0.89 **1.05** 1.17	0.67 **0.89** 1.06	0.80 **0.99** 1.12	F_1,152_ = 17.54^3^***

Mean of Log Afternoon Cortisol Year 3	156	0.29 **0.42** 0.60	0.23 **0.41** 0.55	0.26 **0.42** 0.57	F_1,154_ = 0.99^3^

Mean of Log Evening Cortisol Year 3	157	−0.33 −**0.13** 0.13	−0.31 −**0.10** 0.18	−0.32 −**0.11** 0.17	F_1,155_ = 0.50^3^

CBCL Affective T Scores Year 1	242	50.00 **52.00** 58.67	60.00 **67.00** 73.00	52.00 **60.00** 70.00	F_1,240_ = 114.5^3^***

CBCL Affective T Scores Year 2	171	50.00 **51.00** 56.00	55.17 **63.00** 72.00	50.00 **56.00** 65.00	F_1,169_ = 55.14^3^***

CBCL Affective T Scores Year 3	165	50.00 **51.00** 63.00	51.00 **61.00** 70.00	51.00 **55.00** 66.33	F_1,163_ = 19.12^3^***

*N* is the number of non-missing value.

1 Kruskal-Wallis.

2 Pearson.

3 Wilcoxon.

* *p* ≤ 0.05

** *p* ≤ 0.01

*** *p* ≤ 0.001.

**Table 2.** Diagnosis contrasts for aim 1

Diagnosis contrast conditioned on evening period							
Contrast	Period	Estimate	*SE*	df	*t* ratio	*p*	Bonferroni *p*
ASD – TD	Evening	0.0970	0.0381	208	2.5431	0.0117	NA
**Pairwise contrast between period and diagnosis**							
Contrasts		Estimate	*SE*	df	*t* ratio	*p*	Bonferroni *p*
(Post Waking – Waking ASD) – (Post Waking – Waking TD)		−0.0438	0.0277	3738	−1.5828	0.1135	0.3406
(Afternoon – Post Waking ASD) – (Afternoon – Post Waking TD)		0.1113	0.0268	3738	4.1559	<0.0001	0.0001
(Evening – Post Waking ASD) – (Evening – Post Waking TD)		0.1803	0.041	3738	4.4025	<0.0001	<0.0001

**Table 3.** Age effect and age trend contrasts by period for Hyp 2.1

Age effect						
Effect	Estimate	*SE*	df	*t* value	*p*	Bonferroni *p*
Age	0.0244	0.0059	222	4.1406	< 0.0001	NA
**Age trend contrasts between periods**						
Contrasts	Trend	*SE*	df	*t* ratio	*p*	Bonferroni *p*
Post Waking – Waking	−0.0031	0.0098	3738	− 0.3213	0.7480	1.0000
Afternoon – Post Waking	0.0380	0.0082	3738	4.6431	< 0.0001	< 0.0001
Evening – Post Waking	0.0517	0.0135	3738	3.8202	0.0001	0.0004

*Note.* Age effect is from the model with no age:period interaction.

**Table 4.** Pubertal stage effect & pubertal stage trend contrasts by period for Hyp 2.2

Pubertal stage effect						
Effect	Estimate	*SE*	df	*t* value	*p*	Bonferroni *p*
Pubertal stage	0.0233	0.0067	220	3.4929	0.0006	NA
**Pubertal stage trend contrasts between periods**						
Contrasts	Trend	*SE*	df	*t* ratio	*p*	Bonferroni *p*
Post Waking – Waking	0.0026	0.0097	3711	0.2648	0.7912	1.0000
Afternoon – Post Waking	0.0291	0.0104	3711	2.8118	0.0050	0.0149
Evening – Post Waking	0.0293	0.0151	3711	1.9404	0.0524	0.1572

*Note.* Pubertal stage effect is from the model with no puberty stage: period interaction and no age effect.

**Table 5.** Sex effect and sex contrasts for Hyp 3.1

Sex effect							
Effect		Estimate	*SE*	df	*t* value	*p*	Bonferroni *p*
Sex: female		0.0867	0.0218	208	3.9728	0.0001	NA
**Sex contrast conditioned on evening period**							
Contrast	Period	Estimate	*SE*	df	*t* ratio	*p*	Bonferroni *p*
Female – Male	Evening	0.1766	0.0425	208	4.1526	<0.0001	NA
**Pairwise contrast between period and sex**							
Contrasts		Estimate	*SE*	df	*t* ratio	*p*	Bonferroni *p*
(Post Waking – Waking Female) – (Post Waking – Waking Male)		0.0206	0.0289	3735	0.7124	0.4763	1.0000
(Afternoon – Post Waking Female) – (Afternoon – Post Waking Male)		−0.0170	0.0285	3735	−0.5969	0.5506	1.0000
(Evening – Post Waking Female) – (Evening – Post Waking Male)		0.1069	0.0451	3735	2.3730	0.0177	0.0531

*Note*. Sex effect is from the Hyp 2.1 model.
