# Supplementary material for: The developmental trajectory of diurnal cortisol in autistic and neurotypical youth
Source: Dev Psychopathol. Author manuscript; Available in PMC 2025 Jan 12. (PMC10784411; doi:10.1017/S0954579423000810)
Supplement: 1 [file NIHMS1917005-supplement-1.docx]

**Table S1. Random Effect Standard Deviations with 95% Bootstrap CIs**

| **Random Effects** | **Lower** | **Estimate** | **Upper** |
| --- | --- | --- | --- |
| Day in Year in ID |  |  |  |
| Ratio (ASD/TD) | 0.2887 | 0.7060 | 1.6902 |
| ASD | 0.0084 | 0.0140 | 0.0204 |
| TD | 0.0104 | 0.0226 | 0.0350 |
| Period in ID |  |  |  |
| Ratio (ASD/TD) | 0.9912 | 1.4803 | 2.2027 |
| ASD | 0.0225 | 0.0300 | 0.0374 |
| TD | 0.0149 | 0.0207 | 0.0259 |

**Table S2. Log10 Cortisol Predictors and Random Effects for Hypotheses 1 and 2.1.**

| **Predictors** | **Estimates** | **95% CI** | **p** |
| --- | --- | --- | --- |
| (Intercept) | 0.890 | (0.715, 1.066) | <0.001 |
| Period: Afternoon | -1.017 | (-1.278, -0.757) | <0.001 |
| Period: Evening | -1.782 | (-2.114, -1.449) | <0.001 |
| Period: Post Waking | 0.154 | (-0.083, 0.391) | 0.203 |
| Age | 0.004 | (-0.010, 0.019) | 0.539 |
| Diagnosis: ASD | -0.040 | (-0.091, 0.012) | 0.132 |
| Sex: Female | 0.087 | (0.044, 0.130) | <0.001 |
| BMI | -0.004 | (-0.007, -0.002) | 0.001 |
| Medication: Yes | 0.034 | (-0.004, 0.072) | 0.081 |
| periodAfternoon:Age | 0.035 | (0.013, 0.056) | 0.001 |
| periodEvening:Age | 0.049 | (0.021, 0.076) | <0.001 |
| periodPost Waking:Age | -0.003 | (-0.022, 0.016) | 0.748 |
| periodAfternoon:diagnosisASD | 0.068 | (0.003, 0.132) | 0.041 |
| periodEvening:diagnosisASD | 0.137 | (0.049, 0.224) | 0.002 |
| periodPost Waking:diagnosisASD | -0.044 | (-0.098, 0.010) | 0.114 |
| N ID | 211 |  |  |
| N Year | 3 |  |  |
| N Day | 3 |  |  |
| Observations | 5050 |  |  |
| **Random Effects Standard Deviations** | | | |
| **Random Effects** | **Standard Deviation** | |  |
| ID | 0.082045 | |  |
| Year in ID | 0.106511 | |  |
| Day in Year in ID | 0.054562 | |  |
| Residual | 0.325788 | |  |

**Table S3. Type II ANOVA Table with Period-Age Interaction.**

|  | **X^2^** | **df** | **Pr(>X^2^)** | **Effect Size** |
| --- | --- | --- | --- | --- |
| Period | 2133.718 | 3 | <0.001 | 3.178 |
| Age | 0.764 | 1 | 0.382 | 0.000 |
| Diagnosis | 0.061 | 1 | 0.805 | 0.000 |
| Sex | 8.516 | 1 | 0.004 | 0.189 |
| BMI | 11.929 | 1 | 0.001 | 0.228 |
| Medication | 1.694 | 1 | 0.193 | 0.057 |
| Period:Age | 15.636 | 3 | 0.001 | 0.245 |
| Period:Diagnosis | 12.29 | 3 | 0.006 | 0.210 |

**Table S4. Log10 Cortisol Predictors and Random Effect Model Outputs for Hyp 2.2.**

| **Predictors** | **Estimates** | **95% CI** | **p** |
| --- | --- | --- | --- |
| (Intercept) | 0.931 | (0.869, 0.994) | <0.001 |
| Period: Afternoon | -0.666 | (-0.734, -0.597) | <0.001 |
| Period: Evening | -1.263 | (-1.357, -1.169) | <0.001 |
| Period: Post Waking | 0.107 | (0.054, 0.160) | <0.001 |
| Pubertal Stage | 0.007 | (-0.010, 0.024) | 0.430 |
| Diagnosis: ASD | -0.046 | (-0.098, 0.006) | 0.083 |
| Sex: Female | 0.08 | (0.036, 0.124) | <0.001 |
| BMI | -0.004 | (-0.007, -0.001) | 0.005 |
| Medication: Yes | 0.035 | (-0.004, 0.074) | 0.075 |
| periodAfternoon:Pubertal Stage | 0.032 | (0.006, 0.057) | 0.014 |
| periodEvening:Pubertal Stage | 0.032 | (-0.001, 0.064) | 0.055 |
| periodPost Waking:Pubertal Stage | 0.003 | (-0.016, 0.022) | 0.791 |
| periodAfternoon:diagnosisASD | 0.057 | (-0.007, 0.122) | 0.081 |
| periodEvening:diagnosisASD | 0.125 | (0.037, 0.213) | 0.005 |
| periodPost Waking:diagnosisASD | -0.039 | (-0.094, 0.015) | 0.156 |
| N ID | 210 |  |  |
| N Year | 3 |  |  |
| N Day | 3 |  |  |
| Observations | 5014 |  |  |
| **Random Effects Standard Deviations** | | | |
| **Random Effects** | **Standard Deviation** | |  |
| ID | 0.084215 |  |  |
| Year in ID | 0.107270 |  |  |
| Day in Year in ID | 0.053492 |  |  |
| Residual | 0.326752 |  |  |

**Table S5. Type II ANOVA Table with Period-Puberty Interaction.**

|  | **X^2^** | **df** | **Pr(>X^2^)** | **Effect Size** |
| --- | --- | --- | --- | --- |
| Period | 1749.241 | 3 | <0.001 | 2.884 |
| Pubertal Stage | 5.282 | 1 | 0.022 | 0.143 |
| Diagnosis | 0.118 | 1 | 0.732 | 0.000 |
| Sex | 7.368 | 1 | 0.007 | 0.174 |
| BMI | 6.788 | 1 | 0.009 | 0.166 |
| Medication | 1.860 | 1 | 0.173 | 0.064 |
| Period:Pubertal Stage | 3.890 | 3 | 0.274 | 0.065 |
| Period:Diagnosis | 8.947 | 3 | 0.030 | 0.168 |

**Table S6. Log10 Cortisol Predictors and Random Effect Model Outputs for Hyp 2.3.**

| **Predictors** | **Estimates** | **95% CI** | **p** |
| --- | --- | --- | --- |
| (Intercept) | 0.922 | (0.697, 1.147) | <0.001 |
| Period: Afternoon | -0.954 | (-1.245, -0.662) | <0.001 |
| Period: Evening | -1.807 | (-2.196, -1.418) | <0.001 |
| Period: Post Waking | 0.201 | (-0.084, 0.487) | 0.166 |
| Diagnosis: ASD | -0.043 | (-0.096, 0.010) | 0.113 |
| Pubertal Stage | 0.006 | (-0.018, 0.030) | 0.642 |
| Age | 0.001 | (-0.019, 0.021) | 0.901 |
| Sex: Female | 0.084 | (0.040, 0.127) | <0.001 |
| BMI | -0.005 | (-0.008, -0.001) | 0.004 |
| Medication: Yes | 0.033 | (-0.005, 0.072) | 0.092 |
| periodAfternoon:diagnosisASD | 0.067 | (0.001, 0.134) | 0.047 |
| periodEvening:diagnosisASD | 0.143 | (0.054, 0.231) | 0.002 |
| periodPost Waking:diagnosisASD | -0.043 | (-0.099, 0.013) | 0.132 |
| periodAfternoon:Pubertal Stage | 0.012 | (-0.020, 0.044) | 0.460 |
| periodEvening:Pubertal Stage | -0.005 | (-0.049, 0.039) | 0.820 |
| periodPost Waking:Pubertal Stage | 0.009 | (-0.016, 0.034) | 0.486 |
| periodAfternoon:Age | 0.027 | (0.000, 0.054) | 0.046 |
| periodEvening:Age | 0.051 | (0.015, 0.088) | 0.006 |
| periodPost Waking:Age | -0.009 | (-0.034, 0.017) | 0.497 |
| N ID | 210 |  |  |
| N Year | 3 |  |  |
| N Day | 3 |  |  |
| Observations | 5014 |  |  |
| **Random Effects** | **Standard Deviation** | |  |
| ID | 0.082815 |  |  |
| Year in ID | 0.106555 |  |  |
| Day in Year in ID | 0.055507 |  |  |
| Residual | 0.325628 |  |  |

**Table S7. Type II ANOVA Table with All Interactions.**

|  | **X^2^** | **Df** | **Pr(>X^2^)** | **Effect Size** |
| --- | --- | --- | --- | --- |
| Period | 2202.740 | 3 | <0.001 | 3.237 |
| Diagnosis | 0.033 | 1 | 0.856 | 0.000 |
| Pubertal Stage | 1.295 | 1 | 0.255 | 0.037 |
| Age | 0.527 | 1 | 0.468 | 0.000 |
| Sex | 7.350 | 1 | 0.007 | 0.174 |
| BMI | 7.147 | 1 | 0.008 | 0.171 |
| Medication | 1.433 | 1 | 0.231 | 0.045 |
| Period:Diagnosis | 11.522 | 3 | 0.009 | 0.201 |
| Period:Pubertal Stage | 0.480 | 3 | 0.923 | 0.000 |
| Period:Age | 4.609 | 3 | 0.203 | 0.088 |

**Table S8. Log10 Cortisol Predictors and Random Effect Model Outputs for Hyp 3.1.**

| **Predictors** | **Estimates** | **95% CI** | **p** |
| --- | --- | --- | --- |
| (Intercept) | 0.905 | (0.728, 1.082) | <0.001 |
| Period: Afternoon | -1.018 | (1.279, -0.758) | <0.001 |
| Period: Evening | -1.829 | (-2.161, -1.497) | <0.001 |
| Period: Post Waking | 0.145 | (-0.093, 0.384) | 0.232 |
| Diagnosis: ASD | -0.045 | (-0.097, 0.008) | 0.095 |
| Age | 0.004 | (-0.010, 0.018) | 0.534 |
| Sex: Female | 0.049 | (-0.002, 0.101) | 0.062 |
| BMI | -0.004 | (-0.007, -0.002) | 0.001 |
| Medication: Yes | 0.034 | (-0.004, 0.072) | 0.082 |
| periodAfternoon:diagnosisASD | 0.068 | (0.001, 0.134) | 0.046 |
| periodEvening:diagnosisASD | 0.155 | (0.067, 0.243) | 0.001 |
| periodPost Waking:diagnosisASD | -0.041 | (-0.095, 0.014) | 0.142 |
| periodAfternoon:Age | 0.035 | (0.013, 0.056) | 0.001 |
| periodEvening:Age | 0.048 | (0.022, 0.075) | <0.001 |
| periodPost Waking:Age | -0.003 | (-0.022, 0.016) | 0.751 |
| periodAfternoon:sexFemale | 0.004 | (-0.064, 0.072) | 0.917 |
| periodEvening:sexFemale | 0.128 | (0.032, 0.223) | 0.009 |
| periodPost Waking:sexFemale | 0.021 | (-0.036, 0.077) | 0.476 |
| N ID | 211 |  |  |
| N Year | 3 |  |  |
| N Day | 3 |  |  |
| Observations | 5050 |  |  |
| **Random Effects** | **Standard Deviation** | |  |
| ID | 0.081926 |  |  |
| Year in ID | 0.106538 |  |  |
| Day in Year in ID | 0.056804 |  |  |
| Residual | 0.324645 |  |  |

**Table S9. Type II ANOVA Table with Period-Sex Interaction.**

|  | **X^2^** | **Df** | **Pr(>X^2^)** | **Effect Size** |
| --- | --- | --- | --- | --- |
| Period | 2124.592 | 3 | 0.000 | 3.171 |
| Diagnosis | 0.016 | 1 | 0.900 | 0.000 |
| Age | 0.510 | 1 | 0.475 | 0.000 |
| Sex | 5.159 | 1 | 0.023 | 0.140 |
| BMI | 8.669 | 1 | 0.003 | 0.191 |
| Medication | 1.690 | 1 | 0.194 | 0.057 |
| Period:Diagnosis | 11.080 | 3 | 0.011 | 0.196 |
| Period:Age | 11.703 | 3 | 0.008 | 0.203 |
| Period:Sex | 2.861 | 3 | 0.414 | 0.000 |

**Table S10. Affective Depression Score Predictors and Random Effect Model Outputs for Hyp 3.2.**

| **Predictors** | **Estimates** | **95% CI** | **p** |
| --- | --- | --- | --- |
| (Intercept) | 54.176 | (46.729, 61.624) | <0.001 |
| Evening Mean Cortisol | 0.234 | (-0.486, 0.953) | 0.519 |
| BMI | 0.164 | (-0.172, 0.501) | 0.333 |
| Medication: Yes | 4.519 | (1.240, 7.798) | 0.008 |
| N ID | 69 |  |  |
| Observations | 136 |  |  |
| **Random Effects** | **Standard Deviation** |  |  |
| ID | 7.038648 |  |  |
| Residual | 5.704724 |  |  |
